# Predicting 30-Day Readmissions in an Asian Population: Building a Predictive Model by Incorporating Markers of Hospitalization Severity

**DOI:** 10.1371/journal.pone.0167413

**Published:** 2016-12-09

**Authors:** Lian Leng Low, Nan Liu, Sijia Wang, Julian Thumboo, Marcus Eng Hock Ong, Kheng Hock Lee

**Affiliations:** 1 Department of Family Medicine & Continuing Care, Singapore General Hospital, Singapore; 2 Family Medicine Program, Duke-NUS Medical School, Singapore; 3 Health Services Research Centre, Singapore Health Services, Singapore; 4 Centre for Quantitative Medicine, Duke-NUS Medical School, Singapore; 5 Integrated Health Information Systems, Singapore; 6 Department of Rheumatology and Immunology, Singapore General Hospital, Singapore; 7 Department of Emergency Medicine, Singapore General Hospital, Singapore; 8 Health Services and Systems Research, Duke-NUS Medical School, Singapore; Erasmus Universiteit Rotterdam, NETHERLANDS

## Abstract

**Background:**

To reduce readmissions, it may be cost-effective to consider risk stratification, with targeting intervention programs to patients at high risk of readmissions. In this study, we aimed to derive and validate a prediction model including several novel markers of hospitalization severity, and compare the model with the LACE index (Length of stay, Acuity of admission, Charlson comorbidity index, Emergency department visits in past 6 months), an established risk stratification tool.

**Method:**

This was a retrospective cohort study of all patients ≥ 21 years of age, who were admitted to a tertiary hospital in Singapore from January 1, 2013 through May 31, 2015. Data were extracted from the hospital’s electronic health records. The outcome was defined as unplanned readmissions within 30 days of discharge from the index hospitalization. Candidate predictive variables were broadly grouped into five categories: Patient demographics, social determinants of health, past healthcare utilization, medical comorbidities, and markers of hospitalization severity. Multivariable logistic regression was used to predict the outcome, and receiver operating characteristic analysis was performed to compare our model with the LACE index.

**Results:**

74,102 cases were enrolled for analysis. Of these, 11,492 patient cases (15.5%) were readmitted within 30 days of discharge. A total of fifteen predictive variables were strongly associated with the risk of 30-day readmissions, including number of emergency department visits in the past 6 months, Charlson Comorbidity Index, markers of hospitalization severity such as ‘requiring inpatient dialysis during index admission, and ‘treatment with intravenous furosemide 40 milligrams or more’ during index admission. Our predictive model outperformed the LACE index by achieving larger area under the curve values: 0.78 (95% confidence interval [CI]: 0.77–0.79) versus 0.70 (95% CI: 0.69–0.71).

**Conclusion:**

Several factors are important for the risk of 30-day readmissions, including proxy markers of hospitalization severity.

## Introduction

Preventing unnecessary readmissions is one of the principal challenges facing health systems worldwide. The biggest driver of healthcare cost remains inpatient cost and the average cost of a bed day in Singapore in 2009 was USD 623[1]. The potential cost savings are huge if readmission reduction programs are successful. It has been suggested that reducing unnecessary hospital readmissions first require adequate risk stratification to identify patients at highest risk for readmission, followed by effective intervention programs targeted at modifiable risk factors [2]. It is also widely suggested that readmissions are most preventable in the immediate period after discharge [3]. Therefore, most efforts have concentrated on predicting 30-day readmission risk and developing transitional care programs focused on reducing this risk. Since 2012, hospitals in the United States with excessive 30-day readmission rates for acute myocardial infarction, pneumonia and heart failure are penalized by the Centre for Medicare and Medicaid [4].

For risk stratification, many countries and health systems have developed 30-day readmission models specific to their settings [5], although there is less literature on successful implementation in clinical settings. The more notable prediction scores include PARR-30 developed in the United Kingdom [6], HOSPITAL score [7] in the United States for potentially avoidable readmissions and the LACE index (Length of stay, Acuity of admission, Charlson comorbidity index, Emergency department visits in past 6 months) developed in Ontario, Canada [8]. With the exception of the LACE index, most of these models have limited generalizability to other health systems due to the unique socio-demographic variables [6,9] or have limited clinical utility due to the complexity of the model [5,9,10]. Moreover, the LACE index had moderate discriminative ability *c*-statistic 0.7 despite its simplicity while only four out of 25 other predictive models reviewed by Kansagara et al performed better [5]. The most distinctive advantage of the LACE index is its replicability that allows external validation in populations beyond its original derivation setting. However, to date, the LACE index performed poorly when externally validated in an older UK population with a *c*-statistic 0.55 [11] and in Denmark *c*-statistic 0.648 [12].

Singapore is a city state in South-east Asia with a multi-ethnic population of 5.6 million people. It’s population is one of the most rapidly ageing in Asia with an increasing chronic disease burden [13]. In 2010, the all-cause 30-day readmission rate was 11.6%, but rises to 19.0% in the elderly 65 years and older [14]. This rate is only slightly lower than the 19.6% in the United States [15]. Although Bloomberg rated Singapore as the most efficient healthcare system, healthcare expenditure is expected to triple from S$4 billion in 2011 to S$12 billion in 2020 with 10,000 additional hospital beds required to meet the needs of an aging population. There is great interest to develop a sustainable healthcare system that is future proof for the aging population. Several transitional care programs are already successful in reducing readmission rates [1,16], but these are more criteria driven in patient selection rather than targeted at patients at highest risk of readmission. Consequently, there is a need to identify predictors of readmission risk to derive a predictive model that can guide patient selection for these resource intensive programs.

Suggested predictors of 30-day readmission risk from previous studies include age, Charlson comorbidity index, high-risk medications on discharge, prior healthcare utilization patterns and social determinants of health [5,17–19]. In Kansagara’s review, 14 out of 26 models were derived from retrospective administrative data only and were limited in clinical predictors of illness severity. Only three models looked at real-time automated data and the most promising was Amarasingham et al, who utilized 17 laboratory and vital sign variables. However, missing laboratory and vital sign values would have limited its clinical utility in our setting, especially applied to a general hospital population. In an updated review by Zhou et al on 73 risk predictive models from 2011 to 2015, the most common variables included in predictive models were comorbidities, demographics / social, length of stay and number of previous admissions [20]. A significant gap remained on using markers of hospitalization severity to discriminate for higher risk patients to predict for early 30-day readmissions. Therefore, a potential area of interest in readmission prediction is the markers of hospitalization severity that are discriminative and simple to collect. Any risk score should be available to clinicians and case managers before patient discharge as interventions targeting both the hospitalization phase and post-discharge phase had been found to be most effective [21].

Leveraging on the electronic health record, our primary objective was to systematically investigate known and potential predictors of 30-day readmission risk, including markers of hospitalization severity. The performance of our prediction model will be measured using receiver operating characteristic (ROC) analysis, sensitivity, specificity and precision and compared with the LACE index. We hypothesize that a prediction model incorporating additional markers of hospitalization severity will outperform an established risk stratification tool (the LACE index) at predicting readmissions within 30 days of a patient’s discharge from hospital.

## Methods

### Study design and subjects

This was a retrospective cohort study performed at the Singapore General Hospital (SGH), aiming to compare the LACE index with a predictive model incorporating markers of hospitalization severity. SGH is the largest tertiary hospital in Singapore with 1597 beds, accounting for about 20% of the total public acute hospital beds. SGH is wholly owned by the Ministry of Health Holdings, Singapore. With a workforce numbering above 10,000, SGH admits over 1 million patients every year at its wards, emergency department and outpatient specialist clinics [22].

All admitted adult patients 21 years of age or older from January 1, 2013 through May 31, 2015 at the Singapore General Hospital were enrolled. Patients were eligible for inclusion if they were residents of Singapore. Patients were excluded if they died during the hospitalization or if the admission specialty was obstetrics, emergency medicine, dentistry or ophthalmology. We excluded admissions to the emergency department as these are observation ward stays up to 24 hours duration. We excluded patient cases admitted to obstetrics as these admissions are pregnancy related; and admissions to dentistry and ophthalmology are usually elective in nature. In addition, patients who died during the inpatient admission were excluded.

### Outcome and predictive variables

Clinical and administrative data were extracted from SingHealth’s electronic health records (EHR) system, Electronic Health Intelligence System (eHINTS), which is an enterprise data repository that integrates information from multiple sources, including administrative, clinical and ancillary.

The outcome was defined as unplanned readmissions within 30 days of discharge from the index hospitalization. Readmissions to our hospital were captured by the EHR.

Candidate variables were identified a priori and based on a survey of literatures [5,22–24], and were broadly grouped into five categories: (1) Patient demographics; (2) Social determinants of health; (3) Past healthcare utilization; (4) Medical comorbidities; and (5) Markers of hospitalization severity. Patient demographics included age, gender, and ethnicity. Social determinants of health included the requirement of financial assistance using Medifund (an endowment fund of Singapore government to help needy Singapore citizens to pay the remainder of their hospital bills after receiving government subsidies and drawing on other means of payment including insurance) and admission to a subsidized hospital ward. Past healthcare utilization consisted of number of emergency department (ED) visits in the past six months, number of specialist clinic visits in the past year, and number of hospital admissions in the past year, all before the index admission. For medical comorbidities, chronic diseases such as heart failure, chronic obstructive pulmonary disease, cerebrovascular accident, peripheral vascular disease among other major diseases listed under the Charlson Comorbidity Index, Elixhauser comorbidities and Singapore Ministry of Health Chronic Diseases Program were extracted [25–27]. These diseases were extracted using International Classification of Diseases (ICD) -10 codes of primary and secondary discharge diagnoses dating back to seven years. This is most comprehensive among published literature and would account for potential lapses in diagnostic coding [28]. We also calculated the Charlson Comorbidity Index (CCI) for each patient using the comorbidities.icd10 package in R version 3.2.3 (R Foundation, Vienna, Austria). CCI is also a validated marker for mortality within one year [25].

The psychological state of patients is a well-known factor in coping with illness. We used treatment with anti-depressants in the past one year as a proxy marker of debilitating mood disorders. This is likely to be a more accurate proxy marker than ICD-codes as many mood disorders are managed as outpatients and the use of pharmacy records is recommended to improve validity of ICD coding [29]. For markers of hospitalization severity, we included length of stay of index admission, ‘treatment with intravenous furosemide 40 milligrams or more’; ‘treatment with second line intravenous antibiotics’ (defined as Piperacillin-Tazobactam, Vancomycin, Meropenem and Moxifloxacin); ‘admission to isolation ward’ and ‘required dialysis’. Finally, the LACE index was also computed for each patient [8]. It included four variables, namely length of stay, acute admission, Charlson comorbidity index (CCI), and number of ED visits in the past six months.

### Statistical analysis

We compared the characteristics of patients who were readmitted within 30 days with patients who were not readmitted, using *t*-test for continuous variables and χ^2^ test or Fisher’s exact test for dichotomous variables. We used both univariable and multivariable logistic regressions to examine the associations between candidate predictors and 30-day readmission. The statistical significance was set at p<0.05. In evaluating the predictive models, we adopted 10-fold cross-validation scheme. In 10-fold cross-validation, the whole dataset was first evenly distributed into 10 non-overlapped subsets. This was followed by building a predictive model with nine subsets of data and validated on the remaining one subset. This process was repeated another nine times so that all subsets are tested.

Receiver operating characteristic (ROC) analysis was performed to compare area under the ROC curve (AUC) between our derived regression model and the LACE index. Additionally, sensitivity, specificity, positive predictive value (PPV), and negative predictive value (NPV) were compared and reported. In this study, descriptive analysis and statistical modeling were performed in R version 3.2.3 and the ROC analysis was done in MATLAB R2014b (Mathworks, Natick, MA).

The study was approved by the Singapore Health Services (SingHealth) Centralized Institutional Review Board with waiver of informed consent (CIRB 2015/2696).

## Results

**Fig 1:**
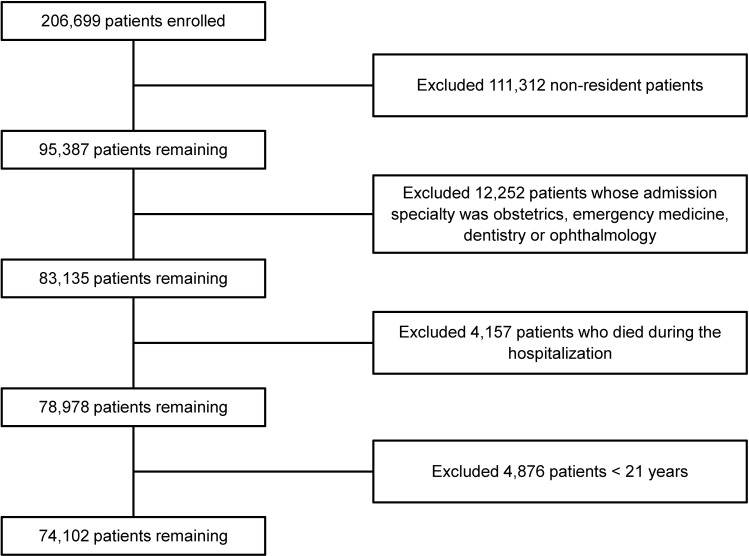
Study flow chart showing number of included, excluded, and readmitted patients.

During the two and a half-year study period, there were 206,699 patient admissions. Of these, 132,597 (64.2%) patient cases were excluded from analysis (Fig 1). The remaining 74,102 patient cases were enrolled with a 30-day readmission rate of 15.5% (n = 11,492). Table 1 presents baseline demographics, prior healthcare utilization, clinical, and comorbidity characteristics of the study population. Patient cases who were readmitted within 30 days after discharge were generally older than patient cases who were not readmitted, with the mean age of 65 (SD = 15) versus 60 (SD = 17). In comparing the readmission group with the non-readmission group, most demographics, clinical and prior healthcare utilization variables were statistically significant except for ‘treatment with second line intravenous antibiotics’ during the inpatient stay (p = 0.947).

**Table 1:** Baseline characteristics by 30-day hospital readmission status.

Variable	All patients (n = 74102)	Readmitted patients (n = 11492)	No readmitted patients (n = 62610)	p-value
**Patient Demographics**				
Age, Mean (SD)	61.27 (17.18)	65.27 (15.57)	60.53 (17.36)	<0.001
Gender, Male (%)	38641 (52.1%)	6374 (55.5%)	32267 (51.5%)	<0.001
Ethnicity				
Chinese (%)	54162 (73.1%)	8667 (75.4%)	45495 (72.7%)	<0.001
Indian (%)	7184 (9.7%)	1091 (9.5%)	6093 (9.7%)	0.438
Malay (%)	9200 (12.4%)	1387 (12.1%)	7813 (12.5%)	0.227
Others (%)	3556 (4.8%)	347 (3%)	3209 (5.1%)	<0.001
Required financial assistance using Medifund (%)	1907 (2.6%)	636 (5.5%)	1271 (2%)	<0.001
**Past Healthcare Utilization**				
ED visits (6 month before index admission), Mean (SD)	1.02 (2.25)	2.48 (4.24)	0.75 (1.49)	<0.001
Specialist Clinic visits (1 year before index admission), Mean (SD)	3.93 (9.19)	6.93 (15.24)	3.37 (7.43)	<0.001
Hospital admissions (1 year before index admission), Mean (SD)	1.58 (3.14)	4.01 (5.88)	1.13 (2.02)	<0.001
**Index Admission Variables**				
Index admission was urgent (%)	57017 (76.9%)	9763 (85%)	47254 (75.5%)	<0.001
Index admission was planned (%)	13471 (18.2%)	1325 (11.5%)	12146 (19.4%)	<0.001
Stayed in a subsidized ward during index admission (%)	60463 (81.6%)	10286 (89.5%)	50177 (80.1%)	<0.001
Required second line antibiotics during index admission (%)	5312 (7.2%)	826 (7.2%)	4486 (7.2%)	0.947
Required inpatient dialysis during index admission (%)	4861 (6.6%)	1233 (10.7%)	3628 (5.8%)	<0.001
Required intravenous Furosemide 40mg and above during index admission (%)	5826 (7.9%)	1268 (11%)	4558 (7.3%)	<0.001
Required isolation during index admission (%)	1422 (1.9%)	300 (2.6%)	1122 (1.8%)	<0.001
Length of stay of index admission, Mean (SD)	6.09 (10.37)	5.29 (5.38)	6.23 (11.04)	<0.001
**Medical Comorbidities (ICD codes past 7 years)**				
Depression (%)	11554 (15.6%)	3396 (29.6%)	8158 (13%)	<0.001
Stroke (%)	955 (1.3%)	146 (1.3%)	809 (1.3%)	0.885
Metastatic Disease (%)	7543 (10.2%)	1244 (10.8%)	6299 (10.1%)	0.013
Non-metastatic malignancy (%)	11810 (15.9%)	1895 (16.5%)	9915 (15.8%)	0.081
Peripheral Vascular Disease (%)	2484 (3.4%)	416 (3.6%)	2068 (3.3%)	0.088
Heart Failure or Fluid Overload (%)	7864 (10.6%)	1236 (10.8%)	6628 (10.6%)	0.6
Pressure Ulcer (%)	1907 (2.6%)	312 (2.7%)	1595 (2.5%)	0.313
Thromboembolism (%)	3896 (5.3%)	628 (5.5%)	3268 (5.2%)	0.29
Spine Fracture (%)	1718 (2.3%)	298 (2.6%)	1420 (2.3%)	0.036
Coronary Heart Disease or Myocardial Infarction (%)	8922 (12%)	1454 (12.7%)	7468 (11.9%)	0.029
Hip Fracture (%)	1199 (1.6%)	185 (1.6%)	1014 (1.6%)	0.971
Atrial Fibrillation (%)	4378 (5.9%)	675 (5.9%)	3703 (5.9%)	0.882
Epilepsy (%)	788 (1.1%)	131 (1.1%)	657 (1%)	0.412
Parkinsonism (%)	840 (1.1%)	123 (1.1%)	717 (1.1%)	0.516
Anxiety (%)	734 (1%)	132 (1.1%)	602 (1%)	0.07
Bipolar Disorder (%)	178 (0.2%)	33 (0.3%)	145 (0.2%)	0.31
Collagen Vascular Disease (%)	1152 (1.6%)	179 (1.6%)	973 (1.6%)	1
Dementia (%)	1840 (2.5%)	256 (2.2%)	1584 (2.5%)	0.06
Hypothyroidism (%)	1323 (1.8%)	216 (1.9%)	1107 (1.8%)	0.429
Chronic Kidney Disease, Stages 1–4 (%)	11266 (15.2%)	1804 (15.7%)	9462 (15.1%)	0.111
Chronic Obstructive Pulmonary Disease (%)	1589 (2.1%)	248 (2.2%)	1341 (2.1%)	0.94
Osteoarthritis (%)	4947 (6.7%)	821 (7.1%)	4126 (6.6%)	0.03
Benign Prostatic Hypertrophy (%)	2651 (3.6%)	382 (3.3%)	2269 (3.6%)	0.118
Asthma (%)	2186 (2.9%)	321 (2.8%)	1865 (3%)	0.294
Hyperlipidemia (%)	17722 (23.9%)	2851 (24.8%)	14871 (23.8%)	0.015
Hypertension (%)	24835 (33.5%)	3925 (34.2%)	20910 (33.4%)	0.117
Chronic Kidney Disease Stage 5 or End Stage Renal Failure (%)	8866(12%)	1441(12.5%)	7425 (11.9%)	0.04
Diabetes (%)	16201 (21.9%)	2614 (22.7%)	13587 (21.7%)	0.013
History of Alcoholism (%)	1372 (1.9%)	316 (2.7%)	1056 (1.7%)	<0.001
Charlson Comorbidity Index, Mean (SD)	2.27 (2.85)	3.83 (3.15)	1.98 (2.69)	<0.001

Readmitted patient cases had used healthcare services more frequently compared with non-readmitted patient cases, in terms of number of ED visits in the past six months (mean = 2.48 [SD = 4.24] versus mean = 0.75 [SD = 1.49]), number of specialist clinic visits in the past year before index admission (mean = 6.93 [SD = 15.24] versus mean = 3.37 [SD = 7.43]), and number of hospital admissions in the past year before index admission (mean = 4.01 [SD = 5.88] versus mean = 1.13 [SD = 2.02]). Medical comorbidities including metastatic disease, spine fracture, coronary heart disease or myocardial infarction, osteoarthritis, hyperlipidemia, chronic kidney disease stage 5 or end stage renal failure, diabetes, history of alcoholism and treatment with anti-depressants in the past one year were statistically significant. Furthermore, readmitted patient cases had a mean CCI of 3.83 and non-readmitted patients had a mean CCI of 1.98.

Univariable and multivariable logistic regressions were performed and the results were shown in Table 2. Several variables were statistically significant in univariable logistic regression but became insignificant in multivariable logistic regression, including number of specialist clinic visits in the past year before index admission, ‘admission to an isolation ward’ during inpatient stay, and the following medical comorbidities (metastatic disease, coronary heart disease or myocardial infarction, hyperlipidemia, chronic kidney disease stage 5 or end stage renal failure, and diabetes).

**Table 2:** Univariable and multivariable logistic regression.

Variable	Unadjusted OR (95% CI)	p-value	Adjusted OR (95% CI)	p-value
**Patient Demographics**				
Age	1.02 (1.02, 1.02)	<0.001	1.01 (1.01, 1.01)	<0.001
Gender (Male)	1.17 (1.13, 1.22)	<0.001	1.09 (1.05, 1.14)	<0.001
Ethnicity				
Others	Baseline			
Chinese	1.15 (1.10, 1.21)	<0.001	1.15 (1.02, 1.31)	0.025
Indian	0.97 (0.91, 1.04)	0.428	0.98 (0.86, 1.14)	0.832
Malay	0.96 (0.91, 1.02)	0.221	1.10 (0.96, 1.26)	0.175
Required financial assistance using Medifund	2.83 (2.56, 3.12)	<0.001	1.24 (1.10, 1.39)	<0.001
**Past Healthcare Utilization**				
ED visits (6 month before index admission)	1.35(1.34, 1.37)	<0.001	1.07 (1.06, 1.08)	<0.001
Specialist Clinic visits (1 year before index admission)	1.04(1.04, 1.04)	<0.001	1.00 (1.00, 1.00)	0.953
Hospital admissions (1 year before index admission)	1.30(1.29, 1.31)	<0.001	1.19 (1.17, 1.20)	<0.001
**Index Admission Variables**				
Index admission was urgent	1.83 (1.74, 1.94)	<0.001	1.49 (1.4, 1.58)	<0.001
Stayed in a subsidized ward during index admission	2.11 (1.99, 2.25)	<0.001	1.43 (1.33, 1.54)	<0.001
Required second line antibiotics during index admission	1.00 (0.93, 1.08)	0.931	1.00 (0.92, 1.10)	0.921
Required inpatient dialysis during index admission	1.95 (1.83, 2.09)	<0.001	1.19 (1.10, 1.29)	<0.001
Required intravenous Furosemide 40mg and above during index admission	1.58 (1.48, 1.69)	<0.001	1.24 (1.15, 1.34)	<0.001
Required isolation during index admission	1.47 (1.29, 1.67)	<0.001	1.15 (0.99, 1.34)	0.069
Length of stay of index admission	0.99 (0.99, 0.99)	<0.001	0.96 (0.96, 0.96)	<0.001
**Medical Comorbidities (ICD codes past 7 years)**				
Depression	2.80 (2.67, 2.93)	<0.001	1.57 (1.49, 1.66)	<0.001
Stroke	0.98 (0.82, 1.17)	0.85	0.92 (0.75, 1.12)	0.432
Metastatic Disease	1.09 (1.02, 1.16)	0.013	1.08 (0.99, 1.18)	0.071
Non-metastatic malignancy	1.10 (0.99, 1.22)	0.083	1.06 (0.93, 1.20)	0.382
Peripheral Vascular Disease	1.02 (0.95, 1.09)	0.588	0.96 (0.87, 1.05)	0.352
Heart Failure or Fluid Overload	1.07 (0.94, 1.21)	0.298	1.09 (0.94, 1.25)	0.237
Pressure Ulcer	1.05 (0.96, 1.15)	0.279	1.01 (0.91, 1.12)	0.859
Thromboembolism	1.05 (0.99, 1.11)	0.078	1.01 (0.94, 1.09)	0.727
Spine Fracture	1.15 (1.01, 1.30)	0.033	1.17 (1.02, 1.35)	0.025
Coronary Heart Disease or Myocardial Infarction	1.07 (1.01, 1.14)	0.028	1.04 (0.96, 1.14)	0.311
Hip Fracture	0.99 (0.85, 1.16)	0.939	0.96 (0.81, 1.14)	0.668
Atrial Fibrillation	0.99 (0.91, 1.08)	0.865	0.98 (0.88, 1.08)	0.628
Epilepsy	1.09 (0.90, 1.31)	0.384	0.97 (0.78, 1.20)	0.794
Parkinsonism	0.93 (0.77, 1.13)	0.486	0.92 (0.74, 1.14)	0.452
Anxiety	1.20 (0.99, 1.44)	0.063	1.17 (0.94, 1.44)	0.147
Bipolar Disorder	1.24 (0.84, 1.79)	0.264	1.31 (0.84, 1.97)	0.217
Collagen Vascular Disease	1.00 (0.85, 1.17)	0.978	0.95 (0.79, 1.13)	0.567
Dementia	0.88 (0.77, 1.00)	0.056	0.89 (0.76, 1.03)	0.117
Hypothyroidism	1.06 (0.92, 1.23)	0.407	1.06 (0.90, 1.26)	0.459
Chronic Kidney Disease, Stages 1–4	1.05 (0.99, 1.11)	0.108	0.98 (0.88, 1.09)	0.652
Chronic Obstructive Pulmonary Disease	1.01 (0.88, 1.15)	0.912	1.00 (0.85, 1.16)	0.961
Osteoarthritis	1.09 (1.01, 1.18)	0.029	1.11 (1.02, 1.22)	0.019
Benign Prostatic Hypertrophy	0.91 (0.82, 1.02)	0.112	0.95 (0.84, 1.07)	0.417
Asthma	0.94 (0.83, 1.05)	0.28	0.87 (0.76, 1.00)	0.055
Hyperlipidemia	1.06 (1.01, 1.11)	0.015	1.03 (0.96, 1.11)	0.395
Hypertension	1.03 (0.99, 1.08)	0.114	0.98 (0.92, 1.06)	0.662
Chronic Kidney Disease Stage 5 or End Stage Renal Failure	1.07 (1.00, 1.13)	0.039	1.04 (0.92, 1.16)	0.551
Diabetes	1.06 (1.01, 1.11)	0.013	1.04 (0.97, 1.12)	0.295
History of Alcoholism	1.65 (1.45, 1.87)	<0.001	1.22 (1.05, 1.42)	0.008
Charlson Comorbidity Index	1.21 (1.20, 1.22)	<0.001	1.14 (1.13,1.15)	<0.001

A total of fifteen predictive variables were strongly associated with the risk of 30-day readmissions (Table 2). We built the multivariable logistic regression model using the following statistically significant variables: age, gender, required financial assistance using Medifund, number of visits to the emergency department in the past 6 months, number of hospital admissions in the past year before index admission, whether or not the index admission was urgent, whether or not staying in a subsidized ward during index admission, required inpatient dialysis during index admission, ‘treatment with intravenous furosemide 40 milligrams or more’ during index admission, length of stay of index admission, comorbidities (depression, spine fracture, osteoarthritis, and history of alcoholism), and the Charlson Comorbidity Index.

**Fig 2:**
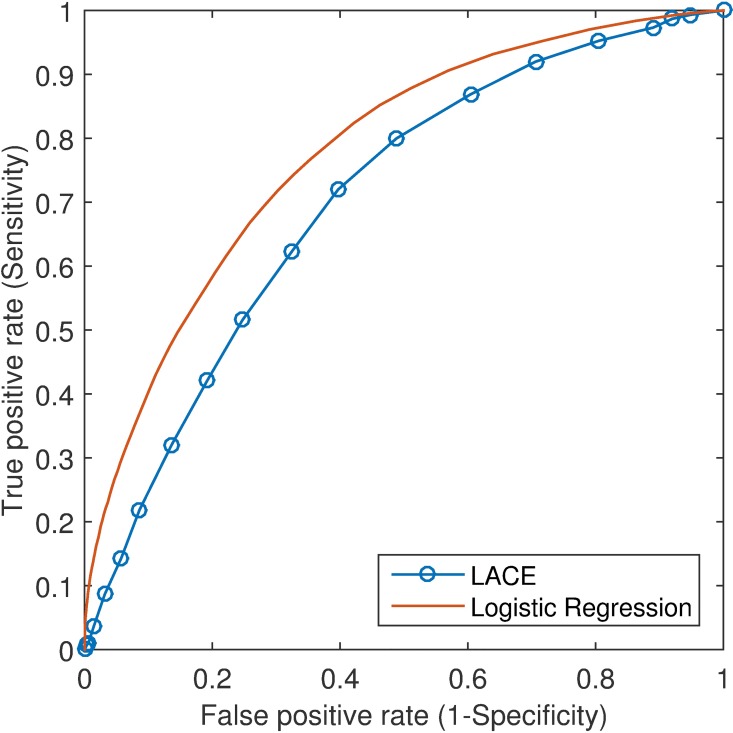
ROC curves of the LACE index and our logistic regression model.

Fig 2 depicted the ROC curves produced by our logistic regression model and the LACE index where the results were based on 10-fold cross-validation. Our model outperformed the LACE index by achieving larger area under the curve (AUC) values: 0.78 (95% confidence interval [CI]: 0.77–0.79) versus 0.70 (95% CI: 0.69–0.71). Moreover, sensitivity, specificity, PPV and NPV were compared where optimal cutoffs were chosen (Table 3). The optimum was determined by the point on the ROC curve, which was nearest to the upper left corner. At the cutoff of 0.14, our logistic regression model obtained sensitivity of 74.3% (95% CI: 73.5%-75.1%) and specificity of 67.3% (95% CI: 66.9%-67.7%). At the cutoff of 8, the LACE index achieved sensitivity of 72.0% (95% CI: 71.2%-72.8%) and specificity of 60.3% (95% CI: 59.9%-60.7%).

**Table 3:** Prediction results by the multivariable logistic regression (LR) model using receiver operating characteristic (ROC) analysis.

	AUC (95% CI)	Cut-off	Sensitivity (95% CI)	Specificity (95% CI)	PPV (95% CI)	NPV (95% CI)
LR	0.78 (0.77–0.79)	0.14	74.3% (73.5% - 75.1%)	67.3% (66.9% - 67.7%)	29.4% (28.9% - 30.0%)	93.4% (93.2% - 93.7%)
LACE	0.70 (0.69–0.71)	8	72.0% (71.2% - 72.8%)	60.3% (59.9% - 60.7%)	25.0% (24.5% - 25.4%)	92.1% (91.9% - 92.4%)

AUC: area under the ROC curve; CI: confidence interval; PPV: positive predictive value; NPV: negative predictive value; LR: logistic regression

**Fig 3:**
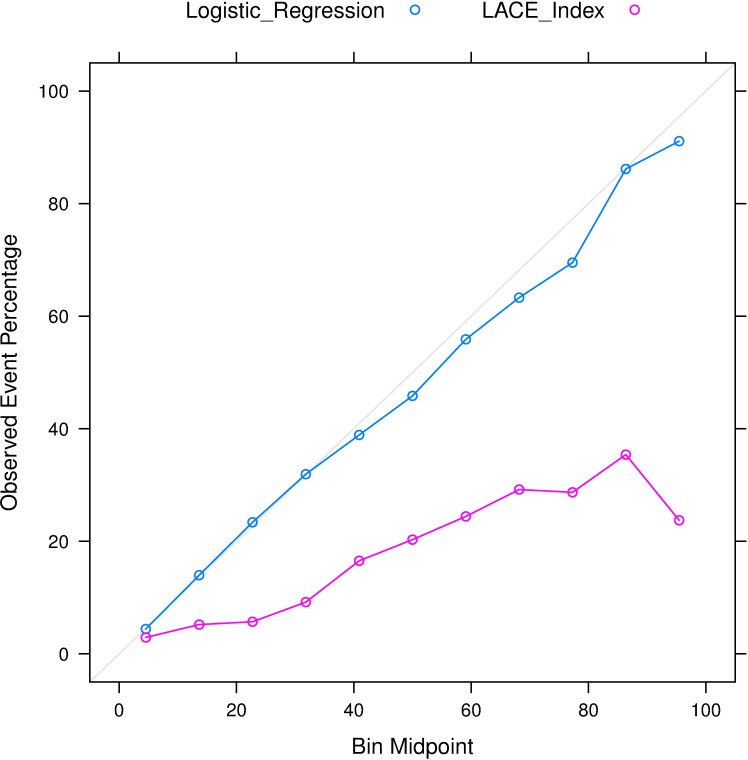
Comparison of the calibration plots between our logistic regression model and the LACE index. The calibration curves were generated using “caret” package in R version 3.2.3 (R Foundation, Vienna, Austria).

Furthermore, the calibration plots (Fig 3) demonstrated that our model achieved stronger agreement between observed and predicted probabilities when compared to the LACE index. In the bin with class probabilities ranging from 20% to 30%, the observed percentage of events for the LACE index was 5.7%, lower than the percentage in our logistic regression model (23.4%). In the bin with class probabilities ranging from 90% to 100%, the observed percentage of events for the LACE index was 23.7%, far lower than the percentage in our model (91.1%).

## Discussion

In this retrospective observational study of 74,102 eligible adult cases, we have identified 15 predictors associated with readmission within 30 days of hospital discharge in Singapore. In this cohort, our predictive model incorporating markers of hospitalization severity and social determinants of health significantly outperformed the LACE index in terms of AUC (0.78 versus 0.70, p<0.001), sensitivity, specificity, positive predictive value and negative predictive value. Compared to published models where majority of the models had an AUC<0.7, our final regression model had good discriminatory power with an AUC of 0.78 and the advantage of being available before patient discharge. We intend to further develop these predictors into a risk score and externally validate the risk score in other health systems. Surprisingly, the performance of the LACE index in our study cohort was comparable to the Ontario population that it was first derived and performed better than the UK and Danish populations that the index was externally validated on [11,12].

Although we started deriving our risk score with 44 variables, we found that 15 factors were important in predicting 30-day readmissions. Our findings that increasing age, prior hospital admissions and emergency department visits and social determinants of health increased 30-day readmission are consistent with existing literature [5,23]. Interestingly, treatment with anti-depressants is the strongest predictor for 30-day readmission risk in our patient cohort. Pederson et al found that one-third of patients discharged from medical wards were depressed [30], while one-sixth of our cohort were treated with anti-depressants. In their review, depressed patients had a 73% higher risk to be readmitted within 30 days compared to 57% in our cohort. Our findings also have implications that depression as a potentially modifiable risk factor could have been under-recognized in risk stratification and under-treated in intervention programs. Similarly, patients with a history of alcoholism may be a surrogate marker of existing or recent social instability. Further investigation on the reasons and causes of 30-day readmissions in these patients would inform the healthcare system to develop focused models of care targeted at these risk factors. Interestingly, increasing length of stay (LOS) was associated with a protective effect for 30-day readmission in our cohort. This is contrary to the findings of Walraven et al and the LACE index. In a review on hospital length of stay and 30-day readmission rate, Kaboli et al found that hospitals with mean risk-adjusted LOS that was lower than expected had a 6% increase in readmission rate for each day lower than expected. These findings and ours suggest that there is a modest trade-off between hospital LOS and readmission, and further investigation is require to examine which patient subgroups are at highest risk of premature discharge and increased risk of adverse outcomes.

Our study is also novel in exploring proxy markers of hospitalization severity on 30-day readmission risk. To our knowledge, no previous studies have evaluated the impact of high risk medications used during the hospitalization stay but instead focused on high risk medications at discharge [17]. Intravenous furosemide is used when prompt and effective diuresis is required and second line antibiotics are used to treat severe hospital acquired infections [31]. Similarly, patients receiving inpatient dialysis could have suffered from acute renal failure or are existing end stage renal failure (ESRF) patients. Although our data does not allow us to differentiate between the two, it is noteworthy that patients with Stage 5 chronic kidney disease or ESRF were not at higher risk of 30-day readmission. We found that ESRF patients requiring inpatient dialysis and patients who received intravenous furosemide 40mg and above were 19% and 24% more likely to be readmitted in the following 30 days. It is possible that undergoing inpatient dialysis and intravenous furosemide better reflect current illness burden than pre-existing medical comorbidities.

In the final multivariable logistic regression model, with the exception of depression, alcoholism and Charlson Comorbidity Index (CCI), the rest of chronic disease comorbidities were not independently associated with 30-day readmission risk. A likely explanation is that comorbidities are confounded by CCI and utilization in past one year. The CCI is a validated measure of one year mortality [25] and covered 11 of the 28 comorbidities investigated in our study. Another explanation is that a patient’s past comorbidities may have lesser significance on his current risk than recent events. Another three (alcoholism, hypothyroidism and parkinsonism) are part of the elixhauser comorbidity index [32], while the rest are from the Singapore chronic disease management program [27]. Our study findings also have implications that tedious retrieval of individual Charlson and Elixhauser comorbidities may not be yielding in determining 30-day risk.

In Kansagara’s review of readmission risk prediction models, only 11 out of the 26 unique models considered social determinants of health [33–35]. Of these 11 models, social determinants of health were defined but not restricted to income, employment status, insurance status, education level and marital status. Social determinants are often unique to the population or country and less generalizable across settings. We selected admission to subsidized ward and requiring medifund for hospitalization bill settlement as social determinants of health in our study, as these information are readily available in our setting. Patients requiring Medifund are among the neediest in Singapore. Medifund is a safety net by the government to reimburse public hospitals for treating patients who would otherwise not afford the hospitalization bill. This ensures no citizen is denied healthcare. Only 2.6% in our patient cohort required financial assistance with Medifund and these patients had a 24% higher risk of 30-day readmission after adjustment for other predictors. Finally, the inclusion or exclusion of these social determinants of health in a risk score should take into consideration the impact on model performance, generalizability and its planned application.

It is noteworthy that many risk predictive models focused on predicting readmissions in cardiovascular-related disease including pneumonia [20,36–41]. As our setting was in a general hospital, we did not restrict our study population to patients with cardiovascular-related disease or pneumonia. In future studies, it would be interesting to validate our findings in this group of patients while incorporating more specific markers of cardiovascular-related disease severity. Unsurprisingly, patients who discharged against medical advice were at higher risk for readmissions in medical and cardiovascular disease related discharges [42,43]. While this indicator is available too late in the admission to include for risk prediction, this group of high-risk patients require attention for post-discharge surveillance.

There is potential to explore other variables of interest in future. These include health literacy, functional status, caregiver availability and markers of social instability [5,44]. At the moment, these data are not routinely collected in most health systems although we have an intention to do so as part of a population database in our hospital. In the interim, we have focused on identifying predictors that can be easily retrieved from a patient’s medical records or can potentially be automated through the EHR. Therefore, we have overlooked variables that require additional collection by healthcare workers currently. Taha et al [45] explored polypharmacy and problem medications such as anti-coagulants and opioids on discharge. While these are available in our EHR system, the discharge prescription is among the finalized documents given to a patient on discharge. Delays in obtaining this information would have limited its usefulness in deriving a risk score for the case managers and clinicians. In deriving our predictors, we have intentionally selected variables that are readily available in the electronic health records (EHR) for a predictive score to be automated or can be easily retrieved from patient medical records and entered into an online spreadsheet or smartphone/tablet application to facilitate clinical use.

### Limitations

Although our study was carefully prepared, several limitations must be considered. Firstly, variables in our dataset are restricted to those routinely collected in the EHR and administrative databases. As such, the granularity of social determinant variables is restricted to ward class and requirement for financial assistance. Functional status, caregiver availability and degree of social support were not routinely collected in our healthcare setting. Secondly, due to the retrospective nature of the study, we were unable to confirm a causal association between the predictor variables and frequent hospital admissions. After our predictive model has identified patients at high risk for 30-day readmission, intervention programs would have to identify potentially modifiable risk factors. Thirdly, we did not exclude patients who might have deceased after index hospital discharge. We felt that that would have biased the prediction model as these patients could have been readmitted before death or died during the readmission. Although data on the 30-day post-discharge mortality rate is not available in Singapore, this outcome only occurred in 0.81% of cases in the Ontario study [8]. Finally, although our study was conducted at the largest health system in Singapore, we were unable to account for readmissions to other health systems. We minimized such bias by excluding patients who are not Singapore citizens or permanent residents.

## Conclusions

In this study we have shown that our predictive model incorporating markers of hospitalization severity and social determinants of health significantly outperformed the LACE index in the ROC analysis. We identified 15 variables that were associated with the risk of 30-day readmission. Among these variables, treatment with anti-depressants was found the strongest predictor, which may have been under-recognized in readmission risk stratification. Furthermore, we explored the use of proxy markers of hospitalization severity in predictive modeling.
